# Heavy metal pollution levels and health risk assessment of dust storms in Jazmurian region, Iran

**DOI:** 10.1038/s41598-023-34318-1

**Published:** 2023-05-05

**Authors:** Mojtaba Soleimani-Sardo, Mahboube Shirani, Vladimir Strezov

**Affiliations:** 1 Department of Environmental Science and Engineering, Faculty of Natural Resources, University of Jiroft, P. O. Box 7867161167, Jiroft, Iran; 2 Department of Chemistry, Faculty of Science, University of Jiroft, P. O. Box 7867161167, Jiroft, Iran; 3 School of Natural Sciences, Faculty of Science and Engineering, Macquarie University, NSW, 2109 Australia

**Keywords:** Ecology, Environmental sciences

## Abstract

The Jazmurian basin in Iran is an area affected by climate change and desertification where aerosols and dust storms are common. The aim of this work was to determine the human and ecological risks from atmospheric particles during dust storms in different cities in the Jazmurian basin. For this purpose, the dust samples were collected from Jiroft, Roodbar Jonoob, Ghaleh Ganj, Kahnooj and Iranshahr cities, which are located around the Jazmurian playa in southeast of Iran. Satellite-based Moderate Resolution Imaging Spectroradiometer (MODIS) aerosol products and the Aerosol Optical Depth (AOD) were used to detect aerosol loading in the atmosphere. Moreover, the trace element composition of the collected particles was determined and used to evaluate human and ecological impact assessment using US EPA human health risk assessment and ReCiPe 2016 endpoint hierarchist impact assessment method incorporated in the OpenLCA 1.10.3 software. The human health risk assessment of the particles revealed high non-carcinogenic risks for children from exposure to nickel and manganese and carcinogenic risks in both adults and children due to hexavalent chromium, arsenic and cobalt during dust storm events. Terrestrial ecotoxicity was found to have the largest ecological impact on ecosystems with copper, nickel and zinc exhibiting the largest contributions.

## Introduction

The arid and semi-arid regions mostly suffer from dust storms as a meteorological phenomenon. Originally, dust storms are the result of gust front or other blowing strong winds in drylands in which both fine and coarse particles are conveyed through suspension where the soil moves from one place and deposits to another^1^. In recent decades, dust storms have caused concerns in Middle Eastern countries due to the increasing trend of climate induced desertification^2^. Dust storms may contain heavy metals and other pollutants which are hazardous to human health^3^. The existence of heavy metals in dust storms is a serious concern owing to the adverse effects with regular exposure and with their deposition in water, soil, all living organisms and foodstuff^4^.

The heavy metals in dust storms can enter the body through ingestion, inhalation and dermal contact^5^ causing irreparable and negative impacts on human health including bronchitis, asthma, lung cancer, respiratory problems, infertility, cardiovascular diseases and nervous system interruptions^6^. The particulate matter (PM) with aerodynamic diameter of less than 10 μm (PM_10_) and 2.5 μm (PM_2.5_) are noticeably important in dust storm studies^7^. High concentrations of PM_10_ and PM_2.5_ can detrimentally impact human respiratory systems and elevate respiratory disorders, such as chronic cough, bronchitis and other chest illnesses^8^. According to the environmental epidemiology research, dust storms are stimulus factors of transmitting and arousing diseases by transporting heavy metals, bacteria and fungi within residents^9^.

In western Asian countries, especially Iran, significant decrease in rainfall has resulted in severe drought and the dry-up of most rivers and lakes^10^. Large parts of the Iran plateau include arid and semi-arid deserts in which aeolian processes are current where mineral dust is often eroded by the wind from dry lands^11^. Jazmurian playa is one of the most significant dryland areas located between the provinces of Sistan Va Baluchestan and Kerman in southeast of Iran, which has changed to a desert over time as a result of climatic variations and human activities, making this area as a high potential region for southeast dust storms in Iran. Jazmuriyan playa is a significant source of dust storms and aeolian sediments in the southeast of Iran and southern Asia due to the morphological, climatological and sedimentological conditions^12^.

The heavy metal pollution in sediments of Jazmurian playa has been a subject of previous study^13^. Jazmurian playa is surrounded by mining activities, which can intensify the heavy metal deposition in the surrounding soils and sediments. Consequently, the dust storms originated in this region can be enriched in toxic heavy metals and their release to the environment can pose significant human health threat. Therefore, analysis of the human health impacts from particles deposited during dust storms in the Juzmurian playa is essential to understand the impact of climate change induced desertification on heavy metal exposure and to assist in developing pollution mitigation strategies. In this study, the dust storm samples from Jazmurian playa were collected from the surrounding cities to evaluate their environmental and human health impacts. Moreover, the average daily intake, carcinogenic risk assessment, and cancer risk were evaluated for the obtained samples.

## Materials and methods description of the study area

Jazmurian playa with a low elevation surface is a seasonal lake located at the center of the Jazmurian basin between the provinces of Sistan Va Baluchestan and Kerman and is covered by aggregations of fine sediments accumulated and deposited with a uniform texture. The Jazmurian depression is surrounded by mountains, Jebal-e Barez in the north and Makran in the south with about 1000 to 3000 m altitude^13^. The average annual rainfall in Jazmurian is more than 200 mm in the northwest altitudes, 150 mm in the eastern part and less than 100 mm in the central part where there is a seasonal lake with a length of 65 km and width of 45 km which sinks into water in winter and then dries out in summer^14^. There is no vegetation in Jazmurian playa because of the severity of aridity and salinity, however poor shrublands cover the adjacent areas. The Jazmurian flat plain and sand dunes are the primary sources of dust aerosols that affect the southeast of Iran and especially the neighboring cities (Jiroft, Kahnooj, Ghaleh Ganj, Iranshahr, Roodbar Jonoob), located away from the region of source.

### Sample collection procedure

**Figure 1:**
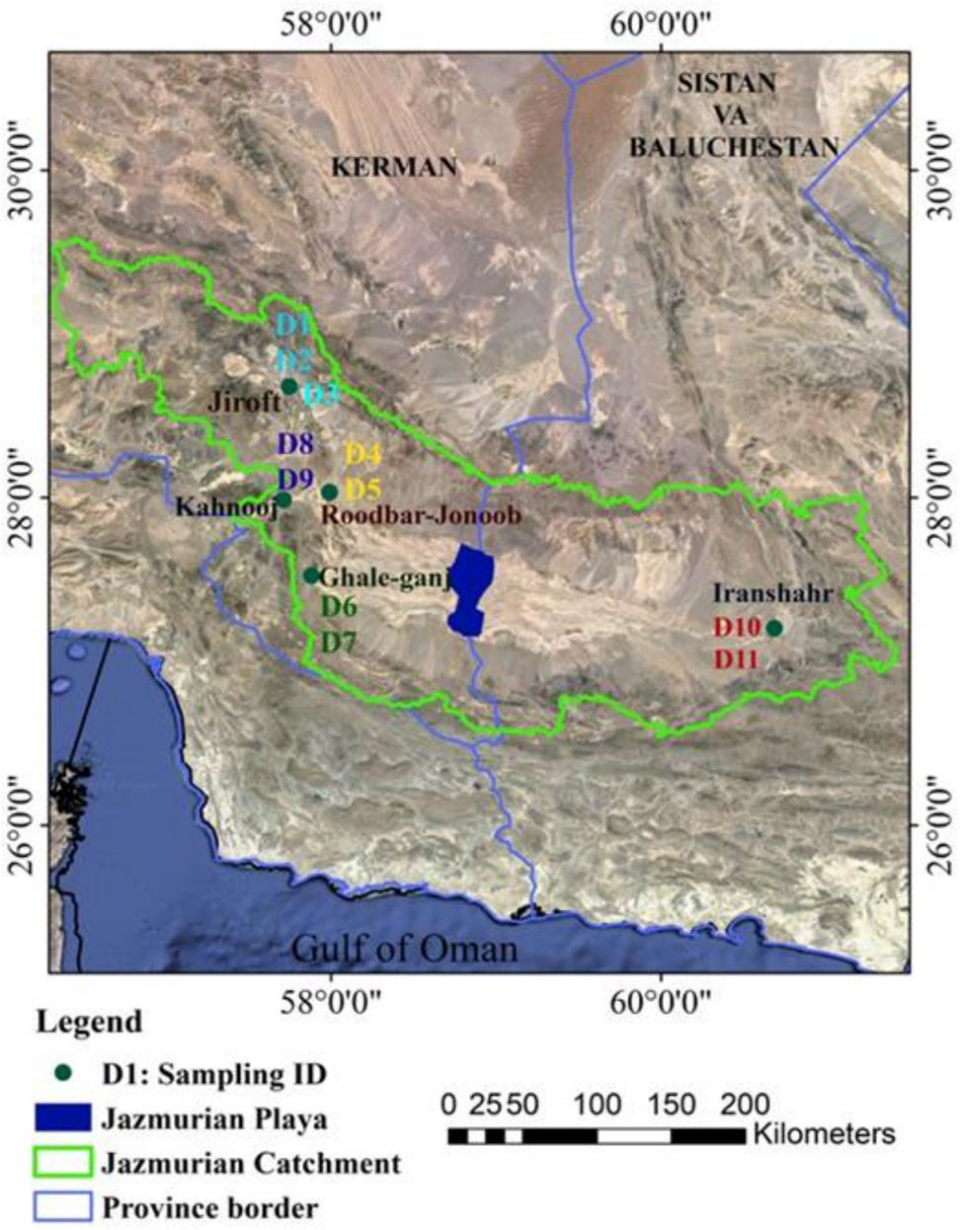
Sampling site for collecting dust samples (Google Earth environment/Data SIO NOAA, US, Navy, NGA, GEBCO/Image Landsat/Copernicus).

In the present study, dust samples from 11 sampling locations were collected with a settleable dust-monitoring device developed in accordance with the ASTM (American Standard Test Method) Standard^15^. The dust samples were collected from Jiroft (Samples D1, D2 and D3), Roodbar Jonoob (D4 and D5) Ghaleh Ganj (D6 and D7), Kahnooj (D8 and D9) and Iranshahr (D10 and D11), which are all located around the Jazmurian playa in southeast of Iran. At each city, about 50 g of dust sample was continuously collected during the dust events from April to August 2021. The samples were placed in clean polyethylene bags, labeled and transported to the laboratory to assess the physical and chemical properties. The sampling locations are shown with a map of the study area in Fig. 1 (Fig. 1 was obtained from the Google Earth environment/ Data SIO NOAA, US, Navy, NGA, GEBCO/Image Landsat/Copernicus. And the coordinates of the data collection stations were placed on it as a point layer by ArcGIS 10.5 software). The concentration of PM_2.5_ and PM_10_ particles for the region during the sampling period were derived from the Kahnooj sampling station managed and reported by the Air Pollution Monitoring System of Iran (available at https://aqms.doe.ir/).

### MODIS aerosol products

Satellite instruments, such as Moderate Resolution Imaging Spectroradiometer (MODIS), have been successfully used to monitor aerosols^16^. In this study, the MODIS Atmosphere L3 Monthly Product from 2001 to 2021 was used. The MODIS Atmosphere L3 Gridded Product is on the basis of Algorithm Theoretical Basis Document (ATBD) (Hubanks et al., 2019). The MODIS aerosol algorithm is described in Kaufman et al. (1997) which provided by the MODIS Characterization Support Team (MCST), identified as products MOD02 and MOD03 for Terra MODIS products (Remer et al., 2005). The Satellite Terra height is about 705 km and it passes the equator at 10:30 am local solar time. MODIS has a high radiometric sensitivity (12 bit) with 36 spectral bands, which makes it possible for monitoring of dust events within a large region.

Aerosol optical depth (AOD) represents aerosol loadings in the atmosphere by calculating their optical depth. MODIS aerosol product prepares daily observations of the AOD over the ocean and land. The MODIS datasets (“Aerosol_Optical_Depth_Land_Ocean_Mean_Mean”: MODIS/061/MOD08_M3) used in this study were provided by NASA LAADS DAAC at NASA Goddard Space Flight Center which is linked to Google Earth Engine Environment. MOD08_M3 V6.1 is an atmosphere global product which contain 1 × 1 degree spatial resolution and roughly 800 statistical datasets that are received from the Level-3 MODIS Atmosphere Daily Global Product^17^ (https://doi.org/10.5067/MODIS/MOD08_M3.061).

### Dust samples preparation for ICP-MS analysis

To prepare the dust samples for ICP-MS analysis, 1 g of each sample was weighed and transferred to a Teflon beaker containing the mixture of acids including: 10 mL HNO_3_, 0.5 mL HCL and 5 mL HF. The solution was dried at 200 °C under vacuum conditions. The sedimented residue was dissolved by the mixture of concentrated HCl: HNO_3_ (1:1 (v/v)) and heated for 5 min to complete the digestion process. The solution was filtered and the filtrate was diluted to 50 mL and then analyzed by ICP-MS (Agilent 7500, USA)^18^.

### QA/QC control

The quality assurance (QA), quality control (QC), and the precision and accuracy of the method were assessed with standard reference materials (SRM) for soils and sediments NIST 2710 and NIST1570A. In each test the blanks and the SRM NIST 2710 were utilized. All obtained data were the mean of three replicates and the standard deviations of blanks were also assessed for three replicates. The samples were spiked at two levels of 25 and 100 µg/g which showed the recoveries in the range of 93.2–102.8% with the relative standard deviation (RSD) values of below 10% which confirm the satisfactory results of precision and accuracy of the analysis method. A total of 63 elements were analysed with the ICP-MS instrument with limit of detection (LOD) of 1 ppm for each element.

### Environmental impact assessment

The elemental analysis of the dust samples and concentration of trace metals were used for environmental impact assessment. In case of chromium the ratio between Cr(VI) and Cr(III) was assumed to be 1:6, hence the total concentration of hexavalent chromium (Cr^6+^) was calculated as 1/7 of the measured total chromium, based on the recommendation by Yan et al.^19^ It has been shown that in a city in Iran up to 25% of the chromium concentrations in atmospheric particles are related to hexavalent chromium^20^. OpenLCA 1.10.3 software was used to input the concentration of the measured trace metals and ReCiPe 2016 endpoint hierarchist impact assessment method^21^ was applied to determine the environmental impacts of each of the dust storm events. The impact assessments considered in this work were for the freshwater ecotoxicity, human carcinogenic toxicity, human non-carcinogenic toxicity, marine ecotoxicity and terrestrial ecotoxicity. The endpoint impact categories were divided into human health impacts and impacts on ecosystems, expressed in disability adjusted life years (DALY) and loss of species during a year (species.yr), respectively.

### Human health risk assessment

The human health risk assessment was determined from the chemical composition of the particles using both human non-carcinogenic and carcinogenic toxicity assessment for adults and children 2–6 years of age. The calculation procedure was adopted based on the US EPA method^22^, which advised that exposure pathway to atmospheric pollutants is based on inhalation. For this reason, only the average daily intake (ADI_inh_) in mg/kg-day of the particles through inhalation, as the major exposure route, was considered in the assessment while the dermal and ingestion exposures to atmospheric particles were not considered. The ADI for each measured trace element *I* was calculated following the formula:

1 $$ ADI_{inh.i} = \frac{{C_{i} \times C_{PM10} /1000 \times InhR \times EF \times ED}}{{BW \times AT \times 10^{6} }} $$

where the C_i_ (mg/kg) is the concentration of trace element i in the particles, C_PM10_ is the reasonable maximum exposure concentration at the upper 95th confidence interval of PM_10_ particles (μg/m^3^) in the atmosphere for the sampling period, calculated in accordance to US EPA^23^ procedure, InhR (m^3^/day) is the inhalation rate, EF (days/year) is the exposure frequency, ED (years) is the exposure duration, BW is the body weight (kg) and AT (days) is the average time period exposed to the trace element. The data used for calculation of ADI was derived from US EPA exposure recommendations^24^ and is shown in Table 1.

**Table 1:** Exposure parameters used to determine the average daily intake of contaminants through inhalation, as determined from US EPA^24^.

Parameter	Unit	Adult	Child
InhR	m^3^/day	15	12.5
EF	days/year	350	350
ED	years	26	6
BW	kg	80	15
AT (non-carcinogenic)	days	9490	2190
AT (carcinogenic)	days	25,550	25,550

For non-carcinogenic toxicity, a hazard quotient (HQ) was determined for each measured trace element as the ratio between the measured ADI_inh.i_ and the reference dose through inhalation for each trace element *i* (RfD_inh.i_):

2 $$ HQ_{i} = ADI_{inh.i} /RfD_{inh.i} $$

The reference dose for each element was determined according to US EPA (ND) and shown in Supplementary Table S1:

3 $$ RfD_{inh.i} = RfC_{i} \frac{InhR}{{BW}} $$

where RfC_i_ (mg/m^3^) is the reference concentration for each trace element derived from US EPA Resident Risk-Based Regional Screening Levels (RSL) for Air online toolkit (https://epa-prgs.ornl.gov/cgi-bin/chemicals/csl_search) and is presented in Supplementary Table S1.

The hazard index was determined as a sum of the estimated hazard quotients:

4 $$ HI = \mathop \sum \limits_{i = 1}^{n} HQ_{i} $$

Non-carcinogenic risks are identified when HI is estimated at greater than 1 with larger risks for greater HI number.

For carcinogenic risk assessment through inhalation of element i, the carcinogenic index CR_inh.i_ is estimated according to:

5 $$ CR_{inh.i} = ADI_{inh.i} \times IUR \times 10^{3} \times \frac{BW}{{InhR}} $$

where IUR (μg/m^3^)^−1^ is inhalation unit risk determined from the US EPA RSL for Air and is displayed in Supplementary Table S1.

The acceptable cancer risk is when the Cancer Risk factor is determined at or lower than 10^−6^, meaning potential cancer risks lower than 1 in a million inhabitants. A range of CR factor between 10^−6^ and 10^−4^ is recommended as a tolerable risk for regulatory purposes^25^.

## Results and discussion

### Aerosol loadings

**Figure 2:**
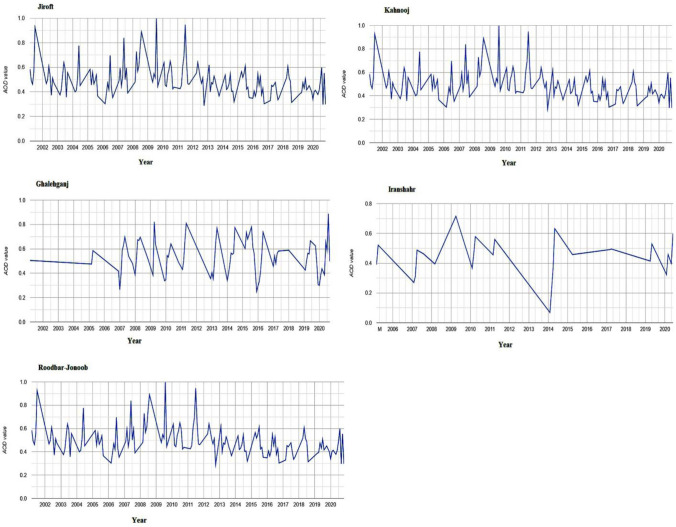
AOD values changes in Jiroft, Kahnooj, Ghalehganj-Iranshshahr and Roodbar jonoob cities.

Atmospheric aerosols resulted from anthropogenic activity (industry, mining) and natural events (dust storms, volcanic ash, smoke) affect human health, reduce visibility and change the Earth’s radiation budget. Aerosols can impact climate change^26^, human health^27^ and ecosystems^28^ through the impacts on air quality^29^. In this study, a time–space distribution and AOD variation over the Jazmurian region was investigated using data from MODIS during the period of 2001–2021 (Fig. 2). 20-years mean of MODIS AOD over the Jazmurian region showed higher values in the south of the basin which gradually decreased to the west, especially for the cities in the west and south of basin (Jiroft, Kahnooj and Ghalehganj) with one in the central part of the basin (Roodbar Jonoob) displaying relatively high AOD of greater than 0.6.

**Figure 3:**
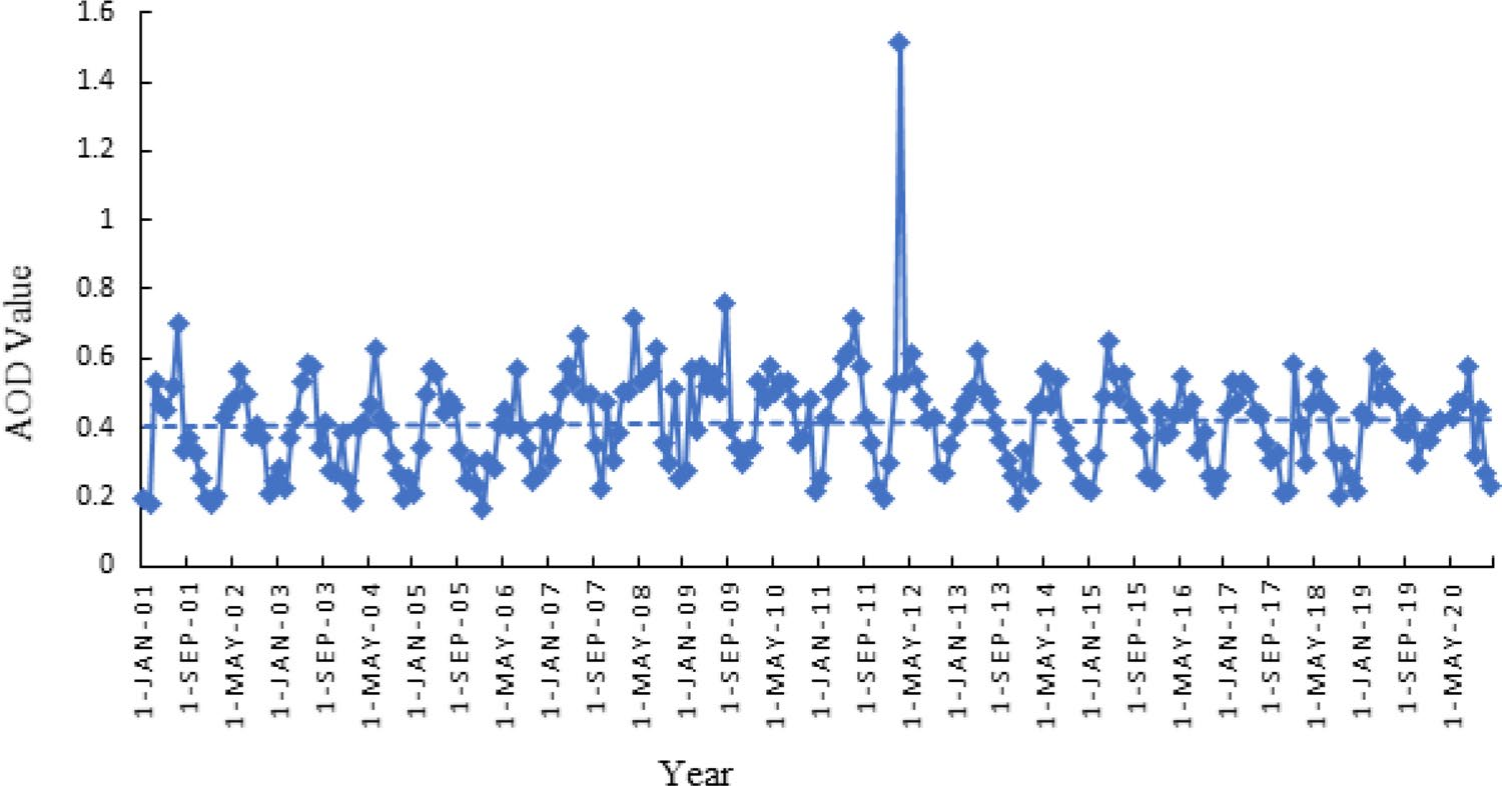
MODIS AOD Values over Jazmurian from 2001–2021.

Drying out of seasonal rivers and surrounding alluvial fans are some of the most important sources of dust in the Jazmurian playa when sediments are exposed to the atmosphere. High MODIS AOD values in the Jazmurian region are also caused by diffusion because of the basin topography and wind speed. Jazmurian region is characterized by a high concentration of desert dust, which is emitted from the Jazmurian playa and the surrounding plains during dust storms observed in Fig. 3 with MODIS AOD values of up to 1.51.

### Characterization of dust storm samples

#### ICP-MS analysis

The chemical analysis of the particles, determined with ICP-MS for all 11 sampling locations is presented in Supplementary Table S2. The results showed large concentrations of trace elements typically encountered in crustal material, such as Fe, Ca, Al, K, Mg and N, indicating the origin of the particles is from the surrounding soils and sediments exposed to atmospheric environment after desertification. Shirani et al.^13^ performed analysis of the sediments in Jazmurian basin and confirmed these elements dominate in the sediments of the area. The other trace elements are determined at variable concentrations at each site.

#### SEM analysis

**Figure 4:**
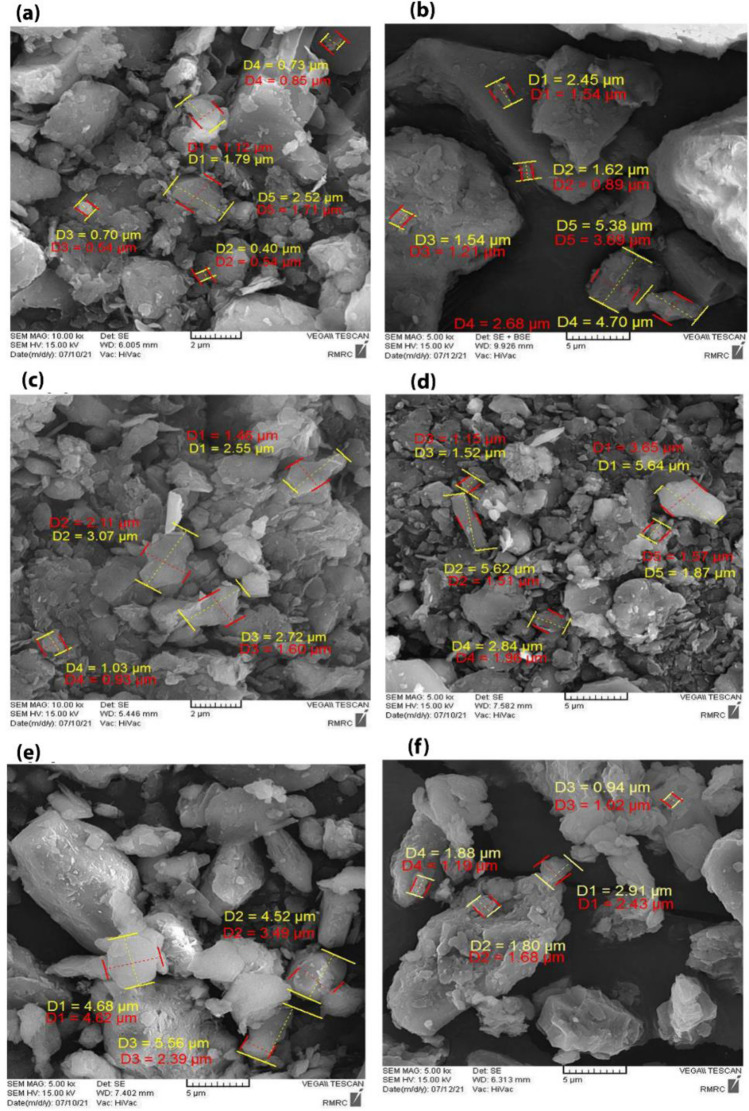
The SEM images of dust samples: (**a**) D1, (**b**) D2, (**c**) D3 (**d**) D4, (**e**) D5, (**f**) D6.

The morphology and size of the dust samples were determined by Field Emission Scanning Electron Microscope (FE-SEM TESCAN MIRA3). Figure 4 shows the SEM images of the collected dust samples with different magnifications. SEM photomicrographs revealed varying shapes and also a heterogeneous mixture of particles of various sizes and morphologies. The difference in particle size and the irregular structural shape of samples detected in the SEM images confirmed the chemical composition variability of the samples. Fine dust particles represent variety in shape, including ellipsoid, spherical and angled shapes. Particles are a mixture of large and small volume particles. Random particle diameters were found to be in the range of 0.40 µm to 5.64 µm according to the SEM image.

#### Impact assessment of the particles

**Figure 5:**
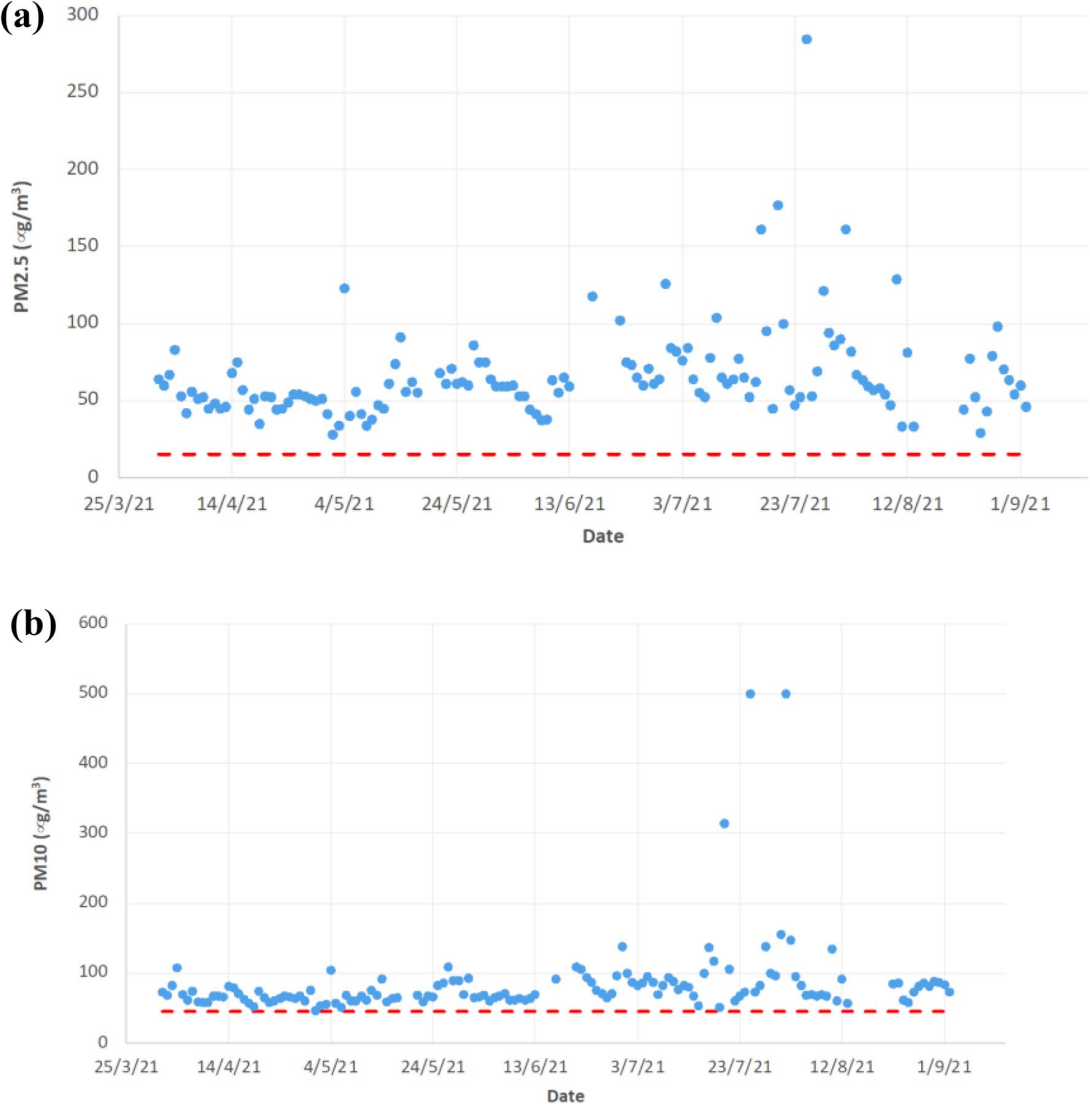
Concentration of atmospheric particulate matter concentrations at Kahnooj sampling station managed and reported by the Air Pollution Monitoring System of Iran; (**a**) PM_2.5_; (**b**) PM_10_. Red dotted lines indicate 24 h average concentrations according to World Health Organisation air quality guidelines (WHO 2021)^29^.

Figure 5 presents the atmospheric PM_2.5_ and PM_10_ concentrations measured at Kahnooj sampling station during the sampling period of the study. The results indicate that the atmospheric concentrations of both PM_2.5_ and PM_10_ particles exceeded the WHO recommended guidelines for 24 h average PM concentrations, which are 15 µg/m^3^ for PM_2.5_ and 45 µg/m^3^ for PM_10_^30^. The average measured concentrations for PM_2.5_ over the sampling period were 66.1 µg/m^3^ and 85.1 µg/m^3^ for PM_2.5_ and PM_10_ respectively, with maximum concentrations reaching 285 µg/m^3^ for PM_2.5_ and 500 µg/m^3^ for PM_10_.

Table 2 shows the results from the environmental impact assessment using the Open LCA software and ReCiPe 2016 endpoint hierarchist impact assessment method. The results show sampling location D11 in Iranshahr region exhibited the largest human health impacts expressed in disability adjusted life years (DALY) due to the impacts of arsenic and zinc on the human non-carcinogenic toxicity and hexavalent chromium on human carcinogenic toxicity. The samples D1, D2 and D3 collected from Jiroft presented human health impacts in a similar range and lower than D11. The other locations had lower environmental impacts with D8 in Kahnooj and D5 in Roodbar Jonoob showing the lowest human health impacts, almost one third of sample D11 in Iranshahr. The ecological impacts, expressed in species extinct per year for each gram of particles, were in the similar range for all samples with sample D8 in Kahnooj demonstrating the largest ecological impacts due to the effect of copper on terrestrial ecotoxicity. Nickel and zinc were the other two contributing factors to the terrestrial ecotoxicity.

**Table 2:** Environmental impact assessment of the dust storm in 11 locations in Jazmurian playa based on their chemical composition. The units are expressed based on 1 g of particles.

		Jiroft			Roodbar Jonoob		Ghaleh-Ganj		Kahnooj		Iranshahr	
		D1	D2	D3	D4	D5	D6	D7	D8	D9	D10	D11
Human carcinogenic toxicity	DALY	7.08∙10^−10^	6.59∙10^−10^	4.53∙10^−10^	6.63∙10^−10^	5.79∙10^−10^	8.42∙10^−10^	5.88∙10^−10^	3.96∙10^−10^	4.69∙10^−10^	7.72∙10^−10^	19.0∙10^−10^
Human non-carcinogenic toxicity	DALY	9.62∙10^−10^	9.62∙10^−10^	9.43∙10^−10^	8.44∙10^−10^	5.90∙10^−10^	6.16∙10^−10^	7.17∙10^−10^	7.38∙10^−10^	7.39∙10^−10^	6.95∙10^−10^	11.2∙10^−10^
Freshwater ecotoxicity	species.yr	1.41∙10^−15^	1.41∙10^−15^	1.57∙10^−15^	1.86∙10^−15^	1.38∙10^−15^	1.63∙10^−15^	1.46∙10^−15^	2.65∙10^−15^	1.78∙10^−15^	1.19∙10^−15^	1.38∙10^−15^
Marine ecotoxicity	species.yr	6.97∙10^−15^	6.96∙10^−15^	7.00∙10^−15^	12.1∙10^−15^	7.53∙10^−15^	6.74∙10^−15^	6.65∙10^−15^	23.1∙10^−15^	10.7∙10^−15^	5.51∙10^−15^	5.76∙10^−15^
Terrestrial ecotoxicity	species.yr	1.43∙10^−12^	1.43∙10^−12^	1.38∙10^−12^	2.73∙10^−12^	1.60∙10^−12^	1.27∙10^−12^	1.29∙10^−12^	5.62∙10^−12^	2.37∙10^−12^	1.11∙10^−12^	1.08∙10^−12^
Human Health Impacts (total)	DALY	16.7∙10^−10^	16.2∙10^−10^	13.9∙10^−10^	15.1∙10^−10^	11.7∙10^−10^	14.6∙10^−10^	13.0∙10^−10^	11.3∙10^−10^	12.1∙10^−10^	14.6∙10^−10^	30.2∙10^−10^
Ecological impacts (total)	species.yr	1.43∙10^−12^	1.43∙10^−12^	1.38∙10^−12^	2.73∙10^−12^	1.60∙10^−12^	1.27∙10^−12^	1.29∙10^−12^	5.62∙10^−12^	2.37∙10^−12^	1.11∙10^−12^	1.08∙10^−12^

Supplementary Tables S3 and S4 present the Average Daily Intake of each of the elements for adults and children used to determine the Hazard Quotient for each element, shown in Supplementary Tables S5 and S6. Table 3 shows the Hazard Index (HI) for each sampling site, estimated as a sum of each individual Hazard Quotients. The Carcinogenic Risk (CR) for both adults and children 2–6 years in Table 3 was estimated from the carcinogenic risk indices of each element with carcinogenic potential, presented in Supplementary Table S7. The results in Table 3 indicate that HI for the adults is close to the threshold of 1, with some sites showing lower values, meaning there may be some minor non-carcinogenic risks to adults in some of the locations. It is in the case of children that the non-carcinogenic effects become apparent, as all locations exhibit HI well above 1 for the children population. Nickel and manganese were the largest contributing factors to non-carcinogenic toxicity in children. The carcinogenic risks were more apparent for all locations as they all exhibited values above the recommended 1∙10^−6^ level, including the recommended tolerable range for regulatory purposes of 1∙10^−6^ to 1∙10^−4^, with children showing significantly higher vulnerability. Hexavalent chromium was the largest contributing factor to carcinogenic toxicity across all sites followed by arsenic and cobalt the highest carcinogenic risks due to the high concentrations of Co comparing to the other locations.

**Table 3:** Human health risk assessment determined for each location.

Location	Sample	HI_adult_	HI_child_	CR_adult_	CR_child_
Jiroft	D1	1.19	5.28	1.33∙10^−4^	1.60∙10^−3^
	D2	1.36	6.05	1.22∙10^−4^	1.46∙10^−3^
	D3	1.38	6.13	0.85∙10^−4^	1.02∙10^−3^
Roodbar Jonoob	D4	1.33	5.91	1.25∙10^−4^	1.49∙10^−3^
	D5	1.23	5.46	1.11∙10^−4^	1.32∙10^−3^
Ghaleh − Ganj	D6	1.06	4.70	1.67∙10^−4^	2.00∙10^−3^
	D7	1.06	4.73	1.14∙10^−4^	1.36∙10^−3^
Kahnooj	D8	0.71	3.19	1.05∙10^−4^	1.26∙10^−3^
	D9	1.11	4.93	0.91∙10^−4^	1.09∙10^−3^
Iranshahr	D10	1.09	4.85	1.59∙10^−4^	1.90∙10^−3^
	D11	1.19	5.28	4.17∙10^−4^	4.99∙10^−3^

Both human health risk assessment methods used in the study, the ReCiPe 2016 presented in Table 2 and US EPA method shown in Table 3 identified hexavalent chromium, arsenic and nickel as the trace elements of largest concern with the US EPA risk assessment method also showing high impacts from cobalt and manganese, while ReCiPe 2016 method also showed zinc as an important trace metal of concern.

According to the results of SEM analysis, the particles size smaller than 2.5 µm was clearly observed which can lead to increased risk of diabetes and penetrate into the lung, corrode the alveolus wall and impair lung function and increase the cardiopulmonary problems and consequently the mortality of lung cancers^31^.

## Conclusions

Jazmurian Playa in Iran is a seasonal lake which experienced desertification in recent times as a result of climate change and human effects. The environmental and human health impacts of the dust particles in the surrounding cities of Jazmurian playa have been investigated in this work.

High value of AOD shown in this work is because of high temperature and evaporation which are significant factors to erosion of soil particles and their movement to the atmosphere by high speed winds. AOD showed a maximum value of 1.514 on 2012 and a minimum value of 0.163 on 2006. Strong winds facilitate the movement of dust aerosols into the air from the dry surface of Jazmurian which can lead to higher AOD. In Jazmurian basin, the Jazmurian lake dried out due to a combination of prolonged drought, dam-building and over-farming, exposing the sediment to atmosphere which contributes to the particle emission during dust storms. The concentrations of both PM_2.5_ and PM_10_ size particles during the five months sampling period exceeded the recommended levels of concentrations by the WHO. The human health risk assessment identified carcinogenic human health risks for children and non-carcinogenic risks for both adults and children. Hexavalent chromium, arsenic and nickel were identified as the most impactful trace elements present in the particles with cobalt, manganese and zinc also posing human health risks. Terrestrial ecotoxicity was the major ecological impact category determined through the impact assessment method used in the study. The study recommends identifying the areas in the Jazmurian basin enriched in chromium, arsenic, nickel, cobalt, manganese and zinc, followed by remedial actions to minimize the risks from exposure through inhalation. Moreover, it suggests that to investigate the source of atmospheric dust particles (internal or external) in the future research to introduce some strategies in order to control erosion in the origin area.

## Supplementary Information

Supplementary Information.

## Data Availability

The datasets generated and/or analysed during the current study are not publicly available due to the regional and national information but are available from the corresponding author on reasonable request.
