# Supplementary material for: Heavy metal pollution levels and health risk assessment of dust storms in Jazmurian region, Iran
Source: Sci Rep. 2023 May 5;13:7337. doi: 10.1038/s41598-023-34318-1 (PMC10163266; doi:10.1038/s41598-023-34318-1)
Supplement: Supplementary file 1 — Supplementary Information. [file 41598_2023_34318_MOESM1_ESM.docx]

**Heavy Metal Pollution Levels and Health Risk Assessment of Dust Storms in Jazmurian Region, Iran**

Mojtaba Soleimani-Sardo^a*^, Mahboube Shirani^b*^, Vladimir Strezov^c^

^a^ Department of Environmental Science and Engineering, Faculty of Natural Resources, University of Jiroft, Jiroft, P. O. Box 7867161167, Iran.

^b^ Department of Chemistry, Faculty of Science, University of Jiroft, Jiroft, P. O. Box 7867161167, Iran.

^c^ School of Natural Sciences, Faculty of Science and Engineering, Macquarie University NSW 2109, Australia.

^*^ Corresponding authors: Tel.: +98 3443347061; fax: +98 34 43347065; Email address: [mojtaba.soleimani@ujiroft.ac.ir](mailto:mojtaba.soleimani@ujiroft.ac.ir); Tel.: +98 3443347061; fax: +98 34 43347065; E-mail address: [m.shirani@ujiroft.ac.ir](mailto:m.shirani@ujiroft.ac.ir)

Table S1. RfC and IUR derived from US EPA Resident Risk-Based Regional Screening Levels (RSL) for Air online toolkit (https://epa-prgs.ornl.gov/cgi-bin/chemicals/csl_search)

|  | RfC (mg/m^3^) | IUR (μg/m^3^)^-1^ |
| --- | --- | --- |
| As | 1.50E-05 | 4.30E-03 |
| Ba | 5.00E-04 |  |
| Be | 2.00E-05 | 2.40E-03 |
| Co | 6.00E-06 | 9.00E-03 |
| Cr(VI) | 1.00E-04 | 8.40E-02 |
| Hg | 3.00E-04 |  |
| Mn | 5.00E-05 |  |
| Ni | 2.00E-05 | 2.60E-04 |
| Sb | 3.00E-04 |  |
| Se | 2.00E-02 |  |
| V | 1.00E-04 |  |

Table S2. Chemical analysis of the particles showing mean values, standard deviations expressed in ppm and relative standard deviation in brackets expressed in %.

|  | Jiroft | | | Roodbar Jonoob | | Ghaleh-Ganj | | Kahnooj | | Iranshahr | |
| --- | --- | --- | --- | --- | --- | --- | --- | --- | --- | --- | --- |
|  | D1 | D2 | D3 | D4 | D5 | D6 | D7 | D8 | D9 | D10 | D11 |
| Ag | <1 | <1 | <1 | <1 | <1 | <1 | <1 | <1 | <1 | <1 | <1 |
| Al | >5000 | >5000 | >5000 | >5000 | >5000 | >5000 | >5000 | >5000 | >5000 | >5000 | >5000 |
| As | 19.0±2.2 (11.5) | 24.3±2.8 (11.7) | 20.5±1.9 (9.3) | 19.2±5.1 (26.8) | 15.2±0.7 (4.6) | 15.1±2.8  (7.0) | 12.7±1.4  (11.2) | 9.3±0.3  (3.7) | 15.5±2.6  (9.3) | 15.2±4.9  (32.3) | 24.3±1.9  (7.7) |
| B | 257.3±3.1 (1.2) | 131.5±2.0 (1.5) | 377.6±7.7 (2.0) | 303.0±5.1 (1.7) | 159.2±3.7 (2.3) | 542.5±8.7  (3.1) | 149.5±19.8  (13.2) | 224.4±1.6  (0.7) | 359.4±5.7  (7.9) | 315.6±13.3  (4.2) | 496.5±8.3  (1.7) |
| Ba | 251.5±2.6 (1.0) | 255.5±5.9 (2.3) | 261.4±30.1 (11.5) | 237.1±2.8 (1.2) | 198.3±4.1 (2.1) | 259.2±6.9  (4.0) | 208.4±7.6  (3.7) | 308.6±0.6  (0.2) | 279.8±6.9  (5.4) | 208.7±9.0  (4.3) | 240±20.5  (8.5) |
| Be | 2.6±0.4 (15.4) | 1.2±0.4 (30.0) | 1.5±0.6 (41.6) | 1.0±0.2 (26.5) | <1 | <1 | 1.0±0.3  (36.1) | 1.3±0.2  (15.4) | <1 | 1.5±0.7  (46.7) | 2.1±0.3  (12.6) |
| Bi | <1 | <1 | <1 | <1 | <1 | <1 | <1 | <1 | <1 | <1 | <1 |
| Ca | >5000 | >5000 | >5000 | >5000 | >5000 | >5000 | >5000 | >5000 | >5000 | >5000 | >5000 |
| Cd | <1 | <1 | <1 | <1 | <1 | <1 | <1 | <1 | <1 | <1 | <1 |
| Ce | 25.6±3.1 (12.3) | 24.7±2.5 (3.1) | 23.7±1.5 (6.4) | 23.5±1.8 (7.8) | 19.5±2.9 (14.8) | 28.7±3.1  (12.1) | 17.2±1.8  (10.3) | 13.7±0.3  (2.6) | 17.0±2.8  (21.5) | 22.3±3.5  (15.9) | 22.3±1.9  (8.6) |
| Co | 5.3±0.6 (11.8) | 10.3±4.2 (41.0) | 4.2±0.2 (6.3) | 6.2±0.6 (10.0) | 5.4±0.4 (8.5) | 10.2±2.5  (6.0) | 3.2±0.5  (17.4) | <1 | 1.1±0.5  (32.8) | 3.4±0.7  (20.6) | 45.5±5.1  (11.3) |
| Cr | 47.1±3.3 (7.1) | 43.3±2.8 (6.4) | 30.3±3.0 (10.2) | 44.3±3.1 (7.0) | 39.3±1.9 (4.9) | 59.1±3.3  (10.1) | 40.6±4.4  (10.9) | 37.5±0.9  (2.3) | 32.5±2.6  (22.9) | 56.2±3.6  (6.5) | 147.4±10.3  (6.8) |
| Cs | 17.2±2.1 (12.6) | 23.8±1.6 (6.9) | 14.4±4.4 (30.4) | 16.2±1.8 (11.5) | 12.1±2.1 (10.6) | 11.5±2.1  (9.2) | 9.4±1.5  (16.4) | 8.4±0.3  (4.1) | 10.4±2.3  (13.6) | 9.1±1.4  (15.5) | 2.3±0.3  (15.1) |
| Cu | 67.5±6.8 (10.0) | 65.5±4.3 (6.6) | 63.5±8.7 (13.8) | 160.7±4.7 (2.9) | 82.0±1.3 (2.7) | 47.2±5.0  (4.9) | 57.2±3.3  (8.7) | 378.3±1.4  (0.4) | 137.2±3.6  11.0 | 48.2±4.3  (9.1) | 44.2±4.3  (9.8) |
| Dy | 9.2±2.3 (25.3) | 13.4±2.6 (19.9) | 9.1±2.4 (26.4) | 10.5±2.4 (23.4) | 7.2±1.4 (19.4) | 8.6±0.8  (12.3) | 6.3±1.4  (12.7) | 5.5±0.3  (5.5) | 5.2±1.1  (8.4) | 6.3±0.7  (11.1) | 2.3±0.4  (19.0) |
| Er | 2.1±0.3 (12.6) | 3.5±1.0 (30.1) | 2.2±0.4 (18.2) | 2.6±1.0 (40.2) | 1.7±0.5 (31.1) | 2.4±0.3  (31.5) | 1.4±0.3  (18.9) | 1.6±0.2  (10.8) | 1.4±0.3  (21.4) | 1.5±0.5  (34.6) | 1.2±0.5  (38.2) |
| Eu | 3.3±0. (8.0) | 4.5±0.6 (14.5) | 3.3±0.5 (9.1) | 3.3±0.2 (8.0) | 2.3±0.5 (19.9) | 2.3±0.6  (23.0) | 2.5±0.5  (24.3) | 1.1±0.4  (32.8) | 2.5±0.7  (16.0) | 2.6±0.4  (16.8) | 1.5±0.4  (24.0) |
| Fe | >5000 | >5000 | >5000 | >5000 | >5000 | >5000 | >5000 | >5000 | >5000 | >5000 | >5000 |
| Ga | 12.5±1.1 (8.9) | 13.5±1.1 (8.2) | 13.4±1.8 (6.8) | 13.4±1.0 (8.0) | 12.2±1.5  (9.7) | 13.5±1.8  (10.3) | 11.6±2.1  (15.5) | 10.5±0.6  (5.9) | 11.5±2.5  (14.0) | 12.3±0.8  (6.3) | 87.3±6.8  (7.8) |
| Gd | 7.1±0.7 (10.1) | 10.3±1.1 (11.0) | 6.5±1.4 (37.8) | 7.5±0.6 (8.3) | 5.5±0.6  (11.4) | 8.7±1.0  (7.5) | 4.4±1.3  (24.1) | 4.3±0.6  (15.2) | 4.5±1.6  (13.5) | 5.5±0.4  (7.3) | 2.6±0.4  (16.8) |
| Ge | 9.7±0.9 (9.1) | 3.2±0.3 (10.8) | 5.1±1.3 (10.4) | 5.5±0.8 (14.4) | 4.8±0.5  (10.8) | 8.4±1.2  (12.4) | 4.3±0.5  (28.2) | 4.7±0.6  (11.3) | 5.6±1.2  (3.1) | 14.1±0.8  (5.8) | 4.1±0.5  (12.7) |
| Hf | 6.4±0.7 (10.9) | 6.4±0.8 (12.8) | 3.2±0.8 (42) | 4.1±0.5 (11.2) | 3.1±0.5  (9.7) | 4.5±0.5  (15.4) | 4.1±0.8  (16.9) | 2.3±0.5  (24.2) | 2.4±0.5  (25.3) | 4.1±1.2  (10.6) | 2.1±0.7  (33.3) |
| Hg | 1.5±0.2 (13.3) | 3.3±0.7 (21.2) | 2.2±0.5 (82.9) | 1.3±0.3 (26.6) | 2.5±0.2  (8.0) | 2.5±0.5  (16.0) | <1 | 1.5±0.3  (20.0) | 2.7±0.6  (22.2) | 1.2±0.4  (8.3) | <1 |
| Ho | 1.3±0.2 (15.4) | 1.2±0.3 (22.0) | 1.2±0.3 (8.3) | 1.5±0.5 (33.3) | 1.4±0.2  (43.4) | 1.3±0.1  (33.5) | 1.1±0.6  (59.6) | <1 | 1.0±0.3  (20.0) | 1.2±0.1  (14.4) | <1 |
| In | <1 | <1 | <1 | <1 | <1 | <1 | <1 | <1 | <1 | <1 | <1 |
| Ir | <1 | <1 | <1 | <1 | <1 | <1 | <1 | <1 | <1 | <1 | <1 |
| K | >5000 | >5000 | >5000 | >5000 | >5000 | >5000 | >5000 | >5000 | >5000 | >5000 | >5000 |
| La | 14.1±1.9 (13.9) | 19.6±3.0 (15.6) | 12.6±2.1 (31.8) | 14.2±1.3 (9.5) | 10.3±2.5  (10.5) | 13.7±2.0  (16.1) | 9.0±1.0  (12.0) | 10.6±0.4  (4.1) | 9.3±1.5  (7.5) | 18.3±1.05  (5.8) | 11.0±2.7  (24.5) |
| Li | 17.0±1.5 (8.9) | 17.3±1.0 (6.1) | 23.3±2.4 (26.0) | 20.3±1.9 (9.40) | 15.5±1.5  (10.6) | 15.3±1.6  (6.6) | 9.1±1.4  (14.4) | 2.3±0.3  (15.1) | 1.6±0.3  (21.7) | 9.3±0.6  (6.5) | 10.2±0.9  (8.5) |
| Lu | 1.0±0.2 (20) | <1 | <1 | <1 | <1 | <1 | <1 | <1 | <1 | <1 | <1 |
| Mg | >5000 | >5000 | >5000 | >5000 | >5000 | >5000 | >5000 | >5000 | >5000 | >5000 | >5000 |
| Mn | 653.5±33.8 (5.1) | 748.5±4.0 (0.5) | 758.6±9.55 (6) | 731.3±5.0 (0.7) | 675.5±3.7  (0.5) | 581.6±4.5  (0.8) | 585.3±27.9  (4.8) | 394.2±4.4  (1.1) | 610.6±3.3  (4.3) | 600.2±30.7  (5.1) | 653.2±3.8  (4.7) |
| Mo | <1 | 1.3±0.2 (13.3) | <1 | <1 | <1 | <1 | <1 | <1 | <1 | <1 | 28.2±1.7  (6.2) |
| Na | >5000 | >5000 | >5000 | >5000 | >5000 | >5000 | >5000 | >5000 | >5000 | >5000 | >5000 |
| Nb | 27.5±2.5 (7.5) | 30.6±2.9 (9.6) | 32.3±2.1 (24) | 68.3±4.3 (6.3) | 16.4±2.2 (15.5) | 24.2±2.2  (5.7) | 17.3±1.2  (7.4) | 21.2±1.8  (8.6) | 23.3±2.1  (10.5) | 29.6±2.4  (8.1) | 15.3±3.5  (9.3) |
| Nd | 41.3±1.6 (3.8) | 52.2±2.3 (4.3) | 33.4±2.6 (21.7) | 38.2±6.7 (17.7) | 29.7±2.6  (10.3) | 45.3±2.3  (5.6) | 23.5±2.8  (11.9) | 22.3±1.9  (8.5) | 24.4±2.0  (9.6) | 27.6±1.5  (5.5) | 20.5±5.1  (16.9) |
| Ni | 64.2±1.7 (2.7) | 63.3±2.1 (3.3) | 24.5±2.0 (10.7) | 58.4±3.4 (5.9) | 49.3±3.3  (7.6) | 64.4±4.8  (7.5) | 53.6±4.5  (8.4) | 16.5±1.1  (6.8) | 29.2±3.1  (6.7) | 37.5±3.7  (9.9) | 30.4±13.3  (16.8) |
| P | 611.6±7.6 (2.5) | 600.5±6.5 (1.1) | 559.6±7.3 (33.9) | 669.5±2.4 (0.3) | 681.5±3.6  (0.5) | 587.6±36.0  (6.1) | 570.4±17.2  (3.0) | 364.4±7.4  (2.0) | 580.3±6.5  (5.0) | 507.5±17.2  (3.4) | 358.2±1.9  (3.7) |
| Pd | 22.3±2.7 (12.0) | 17.5±0.6 (3.7) | 19.4±2.2 (13.4) | 17.5±0.4 (2.0) | 17.3±1.0  (5.7) | 22.5±1.5  (6.8) | 20.3±2.5  (12.2) | 74.4±3.0  (4.0) | 36.2±2.9  (8.2) | 15.3±0.8  (5.1) | 17.3±0.5  (11.5) |
| Pr | 7.3±0.7 (9.6) | 9.3±1.1 (11.6) | 6.3±1.5 (25.5) | 7.6±0.5 (7.3) | 5.2±0.3  (6.9) | 9.5±1.4  (15.2) | 5.3±0.7  (13.6) | 4.5±0.6  (13.5) | 4.3±0.3  (6.2) | 5.3±0.5  (10.5) | 3.8±0.5  (13.9) |
| Re | <1 | <1 | <1 | <1 | <1 | <1 | <1 | <1 | <1 | <1 | <1 |
| Ru | <1 | <1 | <1 | <1 | <1 | <1 | <1 | <1 | <1 | <1 | <1 |
| Rb | 141.3±2.9 (2.0) | 197.4±3.4 (1.7) | 128.5±3.3 (2.0) | 151.5±2.3 (1.5) | 116.3±3.3  (1.4) | 110.2±6.1  (5.6) | 111.0±6.3  (5.7) | 179.3±8.0  (4.5) | 148.7±11.9  (8.0) | 95.6±3.8  (4.0) | 60.6±4.2  (6.9) |
| Sb | 2.2±0.3 (16.4) | 3.3±0.9 (27.7) | <1 | 2.5±0.6 (25.0) | 2.3±0.8  (15.1) | 2.1±0.6  (29.7) | 2.5±0.5  (20.8) | 2.1±0.7  (33.3) | 3.2±0.6  (20.5) | 2.4±0.4  (18.2) | 2.7±0.8  (19.6) |
| Sc | 10.3±1.2 (11.9) | 11.2±1.4 (12.5) | 10.3±2.3 (17.5) | 9.3±1.0 (10.4) | 8.5±1.1  (6.6) | 10.2±0.8  (7.8) | 7.4±1.1  (15.6) | 3.2±0.6  (20.5) | 6.1±0.7  (11.8) | 7.6±0.7  (9.9) | 16.3±1.5  (9.3) |
| Se | 2.5±0.8 (32.7) | 1.4±0.6 (46.8) | <1 | 1.1±0.2 (15.7) | 1.1±0.5  (15.7) | 2.2±0.9  (41.7) | 1.2±0.4  (30.0) | 1.1±0.2  (118.2) | <1 | 1.1±0.7  (68.6) | 2.5±0.4  (16.0) |
| Sm | 50.3±2.3 (4.6) | 6.4±1.0 (16.5) | 4.2±1.5 (25.8) | 4.1±0.3 (8.4) | 3.2±0.5  (8.3) | 4.2±0.4  (8.6) | 3.2±0.5  (17.4) | 2.1±0.8  (40.7) | 3.2±0.6  (8.3) | 3.5±0.6  (17.4) | 4.4±0.5  (11.8) |
| Zr | 37.8±1.2 (3.3) | 35.5±2.0 (5.7) | 33.3±3.2 (13.7) | 36.3±1.0 (2.7) | 34.4±2.1  (7.4) | 44.8±5.1  (11.4) | 40.4±5.3  (13.1) | 17.3±1.5  (8.7) | 26.4±2.1  (10.7) | 40.4±3.2  (7.9) | 34.6±4.1  (12.0) |
| Sn | 23.8±1.5 (6.5) | 16.3±1.5 (9.1) | 1.8±0.2 (16.7) | 7.4±0.8 (10.7) | 17.3±2.3  (3.6) | 5.3±0.5  (10.5) | 3.3±0.5  (13.9) | 2.4±0.6  (25.3) | 12.5±1.4  (11.9) | 60.5±3.8  (5.6) | 3.7±0.5  (14.3 |
| Sr | 320.7±1.5 (0.5) | 299.3±7.7 (2.6) | 325.0±5.5 (18.7) | 286.3±2.0 (0.7) | 295.5±3.5  (1.2) | 272.1±9.0  (3.3) | 260.2±10.7  (4.1) | 197.6±9.4  (4.8) | 320.5±3.3  (5.2) | 272.4±7.8  (2.9) | 254.2±10.4  (4.1) |
| Ta | 12.0±0.8 (6.7) | 12.3±2.5 (20.0) | 6.4±2.2 (10.2) | 10.2±3.0 (30.2) | 6.3±1.6  (4.2) | 8.1±0.7  (9.3) | 7.4±0.8  (11.5) | 9.5±0.9  (10.2) | 7.3±1.8  (5.5) | 7.6±0.6  (8.0) | 5.2±1.3  (6.9) |
| Tb | 1.0±0.2 (25.1) | 1.2±0.2 (14.4) | 1.2±0.5 (25.0) | 1.0±0.4 (36.0) | 1.3±0.4  (27.7) | 1.1±0.4  (41.7) | <1 | <1 | <1 | <1 | <1 |
| Te | 10.5±1.2 (11.1) | 11.5±1.1 (10.0) | 10.4±2.1 (11.1) | 9.3±0.8 (14.8) | 10.5±2.3  (8.5) | <1 | 8.5±1.1  (13.6) | <1 | <1 | <1 | 18.11.3  (7.2) |
| Th | 19.4±1.3 (6.9) | 22.6±1.6 (7.3) | 13.6±2.3 (48.1) | 17.4±0.8 (5.6) | 12.2±2.7  (9.9) | 16.0±2.3  (14.3) | 11.5±1.4  (12.2) | 17.4±0.8  (4.5) | 13.3±0.9  (7.2) | 12.3±1.1  (9.4) | 3.1±0.6  (19.6) |
| Ti | 2335.0±3.5 (0.15) | 2666.1±4.0 (0.1) | 2511.5 (7.7) | 2618.5±7.5 (0.2) | 2277.3±8.7  (0.3) | 2483.4±102.1  (4.1) | 2187.2±69.7  (3.2) | 1336.4±18.2  (1.4) | 1790.4±122.8  (6.9) | 2336.3±65.6  (2.8) | 2118.5±35.7  (1.7) |
| Tl | 1.2±0.3 (25) | 1.0±0.2 (20) | <1 | 1.2±0.3 (22.0) | <1 | <1 | <1 | 1.0±0.6  (62.4) | <1 | <1 | <1 |
| Tm | <1 | <1 | <1 | <1 | <1 | <1 | <1 | <1 | <1 | <1 | <1 |
| U | 3.0±0.4 (13.5) | 3.0±0.4 (12.0) | 2.4±0.6 (52.2) | 3.0±0.4 (14.5) | 2.4±0.6  (15.0) | 3.5±0.5  (15.9) | 2.4±1.0  (45.1) | 2.1±0.5  (25.2) | 3.3±0.2  (6.1) | 2.0±0.6  (31.2) | 1.1±0.3  (27.3) |
| V | 87.5±2.1 (2.4) | 102.5±7.0 (6.9) | 96.3±3.3 (5.8) | 97.6±11.8 (12.1) | 78.5±3.7 (4.8) | 1280.5±94.3  (7.4) | 82.3±10.5  (12.9) | 41.0±3.6  (8.8) | 54.5±7.00  (12.9) | 81.1±7.6  (9.4) | 76.0±8.4  (11.1) |
| W | 6.1±0.5 (9.0) | 9.2±1.0 (10.8) | 5.5±1.3 (16.4) | 5.4±0.3 (4.9) | 44.5±4.2  (9.6) | 6.5±1.2  (21.2) | 6.2±0.9  (14.5) | 6.4±0.4  (7.2) | 6.5±0.4  (6.2) | 8.5±0.5  (6.1) | 3.1±0.4  (12.9) |
| Y | 10.2±0.8 (8.0) | 11.3±1.3 (11.6) | 10.5±2.5 (27.0) | 9.0±0.7 (7.8) | 7.3±1.0  (13.5) | 10.5±0.6  (5.8) | 6.4±0.6  (14.9) | 4.4±0.4  (15.9) | 6.7±0.5  (8.3) | 8.1±0.5  (6.9) | 9.4±0.7  (7.4) |
| Yb | 2.0±0.4 (18.0) | 2.2±0.4 (19.8) | 2.3±0.5 (26.1) | 2.1±0.4 (21.8) | 2.0±0.6  (31.2) | 2.1±0.2  (9.5) | 1.4±0.3  (24.7) | 1.5±0.4  (24.0) | 1.1±0.3  (27.3) | 2.0±0.5  (26.5) | 2.1±0.5  (26.5) |
| Zn | 74.4±2.1 (2.8) | 70.6±3.9 (5.6) | 125.5±8.6 (17.5) | 89.5±3.3 (3.7) | 87.0±1.9  (2.2) | 90.5±7.5  (8.3) | 120.3±10.5  (8.8) | 155.5±4.0  (2.6) | 160.5±12.2  (7.7) | 67.3±8.9  (13.3) | 100.2±4.5  (4.5) |

Table S3. Daily Intake of each element for adults.

|  | D1 | D2 | D3 | D4 | D5 | D6 | D7 | D8 | D9 | D10 | D11 |
| --- | --- | --- | --- | --- | --- | --- | --- | --- | --- | --- | --- |
| As | 3.24E-07 | 4.09E-07 | 3.41E-07 | 3.24E-07 | 2.56E-07 | 2.56E-07 | 2.05E-07 | 1.53E-07 | 2.56E-07 | 2.56E-07 | 4.09E-07 |
| Ba | 4.28E-06 | 4.35E-06 | 4.45E-06 | 4.04E-06 | 3.38E-06 | 4.42E-06 | 3.55E-06 | 5.25E-06 | 4.76E-06 | 3.55E-06 | 4.09E-06 |
| Be | 3.41E-08 | 1.71E-08 | 1.71E-08 | 1.71E-08 |  |  | 1.71E-08 | 1.71E-08 |  | 1.71E-08 | 3.41E-08 |
| Co | 8.53E-08 | 1.71E-07 | 6.82E-08 | 1.02E-07 | 8.53E-08 | 1.71E-07 | 5.12E-08 |  | 1.71E-08 | 5.12E-08 | 7.67E-07 |
| Cr(VI) | 1.15E-07 | 1.05E-07 | 7.31E-08 | 1.07E-07 | 9.50E-08 | 1.44E-07 | 9.74E-08 | 9.01E-08 | 7.80E-08 | 1.36E-07 | 3.58E-07 |
| Cu | 1.14E-06 | 1.11E-06 | 1.07E-06 | 2.73E-06 | 1.40E-06 | 8.02E-07 | 9.72E-07 | 6.45E-06 | 2.34E-06 | 8.19E-07 | 7.50E-07 |
| Hg | 1.71E-08 | 5.12E-08 | 3.41E-08 | 1.71E-08 | 3.41E-08 | 3.41E-08 |  | 1.71E-08 | 3.41E-08 | 1.71E-08 |  |
| Li | 2.90E-07 | 2.90E-07 | 3.92E-07 | 3.41E-07 | 2.56E-07 | 2.56E-07 | 1.53E-07 | 3.41E-08 | 1.88E-07 | 1.53E-07 | 1.71E-07 |
| Mn | 1.11E-05 | 1.28E-05 | 1.29E-05 | 1.25E-05 | 1.15E-05 | 9.91E-06 | 9.98E-06 | 6.72E-06 | 1.04E-05 | 1.02E-05 | 1.11E-05 |
| Ni | 1.09E-06 | 1.07E-06 | 4.09E-07 | 9.89E-07 | 8.36E-07 | 1.09E-06 | 9.04E-07 | 2.73E-07 | 4.95E-07 | 6.31E-07 | 5.12E-07 |
| P | 1.04E-05 | 1.02E-05 | 9.53E-06 | 1.14E-05 | 1.16E-05 | 1.00E-05 | 9.72E-06 | 6.21E-06 | 9.89E-06 | 8.65E-06 | 6.11E-06 |
| Sb | 3.41E-08 | 5.12E-08 |  | 3.41E-08 | 3.41E-08 | 3.41E-08 | 3.41E-08 | 3.41E-08 | 5.12E-08 | 3.41E-08 | 3.41E-08 |
| Se | 3.41E-08 | 1.71E-08 |  | 1.71E-08 | 1.71E-08 | 3.41E-08 | 1.71E-08 | 1.71E-08 |  | 1.71E-08 | 3.41E-08 |
| Sn | 3.92E-07 | 2.73E-07 | 1.71E-08 | 1.19E-07 | 2.90E-07 | 8.53E-08 | 5.12E-08 | 3.41E-08 | 2.05E-07 | 1.02E-06 | 5.12E-08 |
| Sr | 5.46E-06 | 5.10E-06 | 5.54E-06 | 4.88E-06 | 5.03E-06 | 4.64E-06 | 4.43E-06 | 3.36E-06 | 5.46E-06 | 4.64E-06 | 4.33E-06 |
| Th | 3.24E-07 | 3.75E-07 | 2.22E-07 | 2.90E-07 | 2.05E-07 | 2.73E-07 | 1.88E-07 | 2.90E-07 | 2.22E-07 | 2.05E-07 | 5.12E-08 |
| Ti | 3.98E-05 | 4.55E-05 | 4.28E-05 | 4.46E-05 | 3.88E-05 | 4.23E-05 | 3.73E-05 | 2.28E-05 | 3.05E-05 | 3.98E-05 | 3.61E-05 |
| Tl | 1.71E-08 | 1.71E-08 |  | 1.71E-08 |  |  |  | 1.71E-08 |  |  |  |
| U | 5.12E-08 | 5.12E-08 |  | 5.12E-08 |  |  |  |  |  |  |  |
| V | 1.48E-06 | 1.74E-06 | 1.64E-06 | 1.65E-06 | 1.33E-06 | 2.18E-06 | 1.40E-06 | 6.99E-07 | 9.21E-07 | 1.38E-06 | 1.30E-06 |
| Zn | 1.26E-06 | 1.19E-06 | 2.13E-06 | 1.52E-06 | 1.48E-06 | 1.53E-06 | 2.05E-06 | 2.64E-06 | 2.73E-06 | 1.14E-06 | 1.71E-06 |

Table S4. Daily Intake of each element for children.

|  | D1 | D2 | D3 | D4 | D5 | D6 | D7 | D8 | D9 | D10 | D11 |
| --- | --- | --- | --- | --- | --- | --- | --- | --- | --- | --- | --- |
| As | 1.44E-06 | 1.82E-06 | 1.52E-06 | 1.44E-06 | 1.14E-06 | 1.14E-06 | 9.10E-07 | 6.82E-07 | 1.14E-06 | 1.14E-06 | 1.82E-06 |
| Ba | 1.90E-05 | 1.93E-05 | 1.98E-05 | 1.80E-05 | 1.50E-05 | 1.96E-05 | 1.58E-05 | 2.33E-05 | 2.11E-05 | 1.58E-05 | 1.82E-05 |
| Be | 1.52E-07 | 7.58E-08 | 7.58E-08 | 7.58E-08 |  |  | 7.58E-08 | 7.58E-08 |  | 7.58E-08 | 1.52E-07 |
| Co | 3.79E-07 | 7.58E-07 | 3.03E-07 | 4.55E-07 | 3.79E-07 | 7.58E-07 | 2.27E-07 |  | 7.58E-08 | 2.27E-07 | 3.41E-06 |
| Cr(VI) | 5.09E-07 | 4.66E-07 | 3.25E-07 | 4.76E-07 | 4.22E-07 | 6.39E-07 | 4.33E-07 | 4.01E-07 | 3.46E-07 | 6.06E-07 | 1.59E-06 |
| Cu | 5.08E-06 | 4.93E-06 | 4.77E-06 | 1.21E-05 | 6.22E-06 | 3.56E-06 | 4.32E-06 | 2.86E-05 | 1.04E-05 | 3.64E-06 | 3.33E-06 |
| Hg | 7.58E-08 | 2.27E-07 | 1.52E-07 | 7.58E-08 | 1.52E-07 | 1.52E-07 |  | 7.58E-08 | 1.52E-07 | 7.58E-08 |  |
| Li | 1.29E-06 | 1.29E-06 | 1.74E-06 | 1.52E-06 | 1.14E-06 | 1.14E-06 | 6.82E-07 | 1.52E-07 | 8.34E-07 | 6.82E-07 | 7.58E-07 |
| Mn | 4.95E-05 | 5.67E-05 | 5.75E-05 | 5.54E-05 | 5.12E-05 | 4.40E-05 | 4.43E-05 | 2.99E-05 | 4.62E-05 | 4.55E-05 | 4.95E-05 |
| Ni | 4.85E-06 | 4.77E-06 | 1.82E-06 | 4.40E-06 | 3.71E-06 | 4.85E-06 | 4.02E-06 | 1.21E-06 | 2.20E-06 | 2.80E-06 | 2.27E-06 |
| P | 4.63E-05 | 4.55E-05 | 4.24E-05 | 5.07E-05 | 5.16E-05 | 4.45E-05 | 4.32E-05 | 2.76E-05 | 4.40E-05 | 3.84E-05 | 2.71E-05 |
| Sb | 1.52E-07 | 2.27E-07 |  | 1.52E-07 | 1.52E-07 | 1.52E-07 | 1.52E-07 | 1.52E-07 | 2.27E-07 | 1.52E-07 | 1.52E-07 |
| Se | 1.52E-07 | 7.58E-08 |  | 7.58E-08 | 7.58E-08 | 1.52E-07 | 7.58E-08 | 7.58E-08 |  | 7.58E-08 | 1.52E-07 |
| Sn | 1.74E-06 | 1.21E-06 | 7.58E-08 | 5.31E-07 | 1.29E-06 | 3.79E-07 | 2.27E-07 | 1.52E-07 | 9.10E-07 | 4.55E-06 | 2.27E-07 |
| Sr | 2.43E-05 | 2.27E-05 | 2.46E-05 | 2.17E-05 | 2.24E-05 | 2.06E-05 | 1.97E-05 | 1.49E-05 | 2.43E-05 | 2.06E-05 | 1.93E-05 |
| Th | 1.44E-06 | 1.67E-06 | 9.85E-07 | 1.29E-06 | 9.10E-07 | 1.21E-06 | 8.34E-07 | 1.29E-06 | 9.85E-07 | 9.10E-07 | 2.27E-07 |
| Ti | 1.77E-04 | 2.02E-04 | 1.90E-04 | 1.98E-04 | 1.73E-04 | 1.88E-04 | 1.66E-04 | 1.01E-04 | 1.36E-04 | 1.77E-04 | 1.61E-04 |
| Tl | 7.58E-08 | 7.58E-08 |  | 7.58E-08 |  |  |  | 7.58E-08 |  |  |  |
| U | 2.27E-07 | 2.27E-07 |  | 2.27E-07 |  |  |  |  |  |  |  |
| V | 6.59E-06 | 7.73E-06 | 7.28E-06 | 7.35E-06 | 5.91E-06 | 9.70E-06 | 6.22E-06 | 3.11E-06 | 4.09E-06 | 6.14E-06 | 5.76E-06 |
| Zn | 5.61E-06 | 5.31E-06 | 9.47E-06 | 6.75E-06 | 6.59E-06 | 6.82E-06 | 9.10E-06 | 1.17E-05 | 1.21E-05 | 5.08E-06 | 7.58E-06 |

Table S5. Hazard Quotient of each element for adults.

|  | D1 | D2 | D3 | D4 | D5 | D6 | D7 | D8 | D9 | D10 | D11 |
| --- | --- | --- | --- | --- | --- | --- | --- | --- | --- | --- | --- |
| As | 1.15E-01 | 1.46E-01 | 1.21E-01 | 1.15E-01 | 9.10E-02 | 9.10E-02 | 7.28E-02 | 5.46E-02 | 9.10E-02 | 9.10E-02 | 1.46E-01 |
| Ba | 4.57E-02 | 4.64E-02 | 4.75E-02 | 4.31E-02 | 3.60E-02 | 4.71E-02 | 3.78E-02 | 5.60E-02 | 5.08E-02 | 3.78E-02 | 4.37E-02 |
| Be | 9.10E-03 | 4.55E-03 | 4.55E-03 | 4.55E-03 |  |  | 4.55E-03 | 4.55E-03 |  | 4.55E-03 | 9.10E-03 |
| Co | 7.58E-02 | 1.52E-01 | 6.06E-02 | 9.10E-02 | 7.58E-02 | 1.52E-01 | 4.55E-02 |  | 1.52E-02 | 4.55E-02 | 6.82E-01 |
| Cr(VI) | 6.11E-03 | 5.59E-03 | 3.90E-03 | 5.72E-03 | 5.07E-03 | 7.67E-03 | 5.20E-03 | 4.81E-03 | 4.16E-03 | 7.28E-03 | 1.91E-02 |
| Hg | 3.03E-04 | 9.10E-04 | 6.06E-04 | 3.03E-04 | 6.06E-04 | 6.06E-04 |  | 3.03E-04 | 6.06E-04 | 3.03E-04 |  |
| Mn | 1.19E+00 | 1.36E+00 | 1.38E+00 | 1.33E+00 | 1.23E+00 | 1.06E+00 | 1.06E+00 | 7.17E-01 | 1.11E+00 | 1.09E+00 | 1.19E+00 |
| Ni | 2.91E-01 | 2.86E-01 | 1.09E-01 | 2.64E-01 | 2.23E-01 | 2.91E-01 | 2.41E-01 | 7.28E-02 | 1.32E-01 | 1.68E-01 | 1.36E-01 |
| Sb | 6.06E-04 | 9.10E-04 |  | 6.06E-04 | 6.06E-04 | 6.06E-04 | 6.06E-04 | 6.06E-04 | 9.10E-04 | 6.06E-04 | 6.06E-04 |
| Se | 9.10E-06 | 4.55E-06 |  | 4.55E-06 | 4.55E-06 | 9.10E-06 | 4.55E-06 | 4.55E-06 |  | 4.55E-06 | 9.10E-06 |

Table S6. Hazard Quotient of each element for children.

|  | D1 | D2 | D3 | D4 | D5 | D6 | D7 | D8 | D9 | D10 | D11 |
| --- | --- | --- | --- | --- | --- | --- | --- | --- | --- | --- | --- |
| As | 0.51 | 0.65 | 0.54 | 0.51 | 0.40 | 0.40 | 0.32 | 0.24 | 0.40 | 0.40 | 0.65 |
| Ba | 0.20 | 0.21 | 0.21 | 0.19 | 0.16 | 0.21 | 0.17 | 0.25 | 0.23 | 0.17 | 0.19 |
| Be | 0.04 | 0.02 | 0.02 | 0.02 |  |  | 0.02 | 0.02 |  | 0.02 | 0.04 |
| Co | 0.34 | 0.67 | 0.27 | 0.40 | 0.34 | 0.67 | 0.20 |  | 0.07 | 0.20 | 3.03 |
| Cr(VI) | 0.03 | 0.02 | 0.02 | 0.03 | 0.02 | 0.03 | 0.02 | 0.02 | 0.02 | 0.03 | 0.08 |
| Hg | 0.001 | 0.004 | 0.003 | 0.001 | 0.003 | 0.003 |  | 0.001 | 0.003 | 0.001 |  |
| Mn | 5.28 | 6.05 | 6.13 | 5.91 | 5.46 | 4.70 | 4.73 | 3.19 | 4.93 | 4.85 | 5.28 |
| Ni | 1.29 | 1.27 | 0.49 | 1.17 | 0.99 | 1.29 | 1.07 | 0.32 | 0.59 | 0.75 | 0.61 |
| Sb | 2.69E-03 | 4.04E-03 |  | 2.69E-03 | 2.69E-03 | 2.69E-03 | 2.69E-03 | 2.69E-03 | 4.04E-03 | 2.69E-03 | 2.69E-03 |
| Se | 4.04E-05 | 2.02E-05 |  | 2.02E-05 | 2.02E-05 | 4.04E-05 | 2.02E-05 | 2.02E-05 |  | 2.02E-05 | 4.04E-05 |

Table S7. Carcinogenic Risk index of each element for adults and children.

| CR for Adults | |  |  |  |  |  |  |  |  |  |  |
| --- | --- | --- | --- | --- | --- | --- | --- | --- | --- | --- | --- |
|  | D1 | D2 | D3 | D4 | D5 | D6 | D7 | D8 | D9 | D10 | D11 |
| As | 2.76E-06 | 3.49E-06 | 2.91E-06 | 2.76E-06 | 2.18E-06 | 2.18E-06 | 1.74E-06 | 1.31E-06 | 2.18E-06 | 2.18E-06 | 3.49E-06 |
| Be | 1.62E-07 | 8.11E-08 | 8.11E-08 | 8.11E-08 |  |  | 8.11E-08 | 8.11E-08 |  | 8.11E-08 | 1.62E-07 |
| Co | 1.52E-06 | 3.04E-06 | 1.22E-06 | 1.82E-06 | 1.52E-06 | 3.04E-06 | 9.12E-07 |  | 3.04E-07 | 9.12E-07 | 1.37E-05 |
| Cr(VI) | 1.33E-04 | 1.22E-04 | 8.51E-05 | 1.25E-04 | 1.11E-04 | 1.67E-04 | 1.14E-04 | 1.05E-04 | 9.08E-05 | 1.59E-04 | 4.17E-04 |
| Ni | 5.62E-07 | 5.53E-07 | 2.11E-07 | 5.09E-07 | 4.30E-07 | 5.62E-07 | 4.66E-07 | 1.41E-07 | 2.55E-07 | 3.25E-07 | 2.64E-07 |
|  |  |  |  |  |  |  |  |  |  |  |  |
| CR for children | |  |  |  |  |  |  |  |  |  |  |
| As | 3.30E-05 | 4.17E-05 | 3.48E-05 | 3.30E-05 | 2.61E-05 | 2.61E-05 | 2.09E-05 | 1.56E-05 | 2.61E-05 | 2.61E-05 | 4.17E-05 |
| Be | 1.94E-06 | 9.70E-07 | 9.70E-07 | 9.70E-07 |  |  | 9.70E-07 | 9.70E-07 |  | 9.70E-07 | 1.94E-06 |
| Co | 1.82E-05 | 3.64E-05 | 1.46E-05 | 2.18E-05 | 1.82E-05 | 3.64E-05 | 1.09E-05 |  | 3.64E-06 | 1.09E-05 | 1.64E-04 |
| Cr(VI) | 1.60E-03 | 1.46E-03 | 1.02E-03 | 1.49E-03 | 1.32E-03 | 2.00E-03 | 1.36E-03 | 1.26E-03 | 1.09E-03 | 1.90E-03 | 4.99E-03 |
| Ni | 6.73E-06 | 6.62E-06 | 2.52E-06 | 6.10E-06 | 5.15E-06 | 6.73E-06 | 5.57E-06 | 1.68E-06 | 3.05E-06 | 3.89E-06 | 3.15E-06 |
